# Role of transient receptor potential channels in regulating spermatozoa functions: A mini-review

**DOI:** 10.14202/vetworld.2018.1618-1623

**Published:** 2018-11-23

**Authors:** Akshay Kumar, Abhishek Kumar Mishra, Dilip Kumar Swain, Vijay Singh, Sarvajeet Yadav, Atul Saxena

**Affiliations:** 1 Department of Gynaecology and Obstetrics, College of Veterinary Sciences and Animal Husbandry, U.P. Pandit Deen Dayal Upadhyaya Pashu Chikitsa Vigyan Vishwavidyalaya Evam Go Anusandhan Sansthan, Mathura, Uttar Pradesh, India; 2 College of Biotechnology, U.P. Pandit Deen Dayal Upadhyaya Pashu Chikitsa Vigyan Vishwavidyalaya Evam Go Anusandhan Sansthan, Mathura, Uttar Pradesh, India; 3 Department of Physiology, College of Veterinary Sciences and Animal Husbandry, U.P. Pandit Deen Dayal Upadhyaya Pashu Chikitsa Vigyan Vishwavidyalaya Evam Go Anusandhan Sansthan, Mathura, Uttar Pradesh, India

**Keywords:** acrosome reaction, calcium, capacitation, fertilization, motility, pH, sperm, temperature, transient receptor potential

## Abstract

Flagellar navigation along the genital tract of male and female in spermatozoa is accomplished through a number of biological, physiological, biochemical, and electrophysiological alterations in spermatozoa. These alterations are highly precise, dynamic, and regulated through a number of ion channels along with their associated pathways. Beating of flagella along with intracellular metabolism of spermatozoa is associated with fluxing of Ca++ as well as release of Ca++ from different sources. Calcium fluxing through the spermatozoa is mediated through sperm-specific calcium channel and also through transient receptor potential (TRP) channels which are diversified multifamily of ion channels which are activated through a number of extracellular agents such as pH, temperature, chemicals, and pathogens. Research has shown the dynamic role of TRP channels in regulating sperm functions such as sperm chemotaxis, rheotaxis, thermotaxis, and eventually fertilization. Diversified forms of TRP and their involvement in regulation of sperm function opens new horizons of understanding of the sperm function and, in specific, issues related to infertility. This mini-review is an attempt to draw some insights into the action of TRP channels in regulating sperm fertility competence through both calcium-dependent and calcium-independent mechanisms.

## Introduction

Spermatozoa navigation in different chemical, physical, and fluid gradients is very complex requiring controlled and precise role of ion channels along with their downstream signal transduction pathways. Ion channels are membrane proteins being present in all living cells including sperm cells and are tightly regulated by a number of extracellular and intracellular factors [1]. From ancient times, it was believed that these are confined to excitable cells such as neurons and myocytes, but studies have shown the presence of ion channels in sperm cells and their involvement in regulation of numerous functions of sperm cells. In the recent past, the emergence of sperm ion channels has revolutionized modern understanding of sperm function and, in specific, sperm functions such as motility, capacitation, chemotaxis, and acrosomal reaction [2].

Flagellated spermatozoa exhibit kinetic motion to spot the egg to bring out fertilization which is governed through a number of intrinsic as well as extrinsic factors [3]. Key events of sperm motion in different chemical, physical, and fluid gradients are accomplished through Ca^2+^ and its associated signaling mechanisms [3]. Regulation of Ca^2+^ influx is governed through sperm-specific Ca^2+^ channels (CatSper) and other ion channels such as transient receptor potentials (TRPs) [2]. Species not having defined CatSper channels also exhibit Ca^2+^ influx and therefore hint toward the existence of other sets of mechanisms to regulate fluxing of Ca^2+^ into sperm cells [2]. TRP channels are large set of cation channels and regulate Ca^2+^ influx as well as release of Ca^2+^ from storage sites in spermatozoa [4].

Sperm cells are considered as dynamic cells and are the only cells to encounter their total lifespan in different physiological conditions starting from testicular microenvironment to male reproductive tract and after ejaculation in female genital tract [5,6]. It is very interesting to understand that how spermatozoa function in such altered and diverse environments. Sperm cells are regulated by array of fast crosstalk mechanisms and are believed to be regulated through ion channels [5,6]. Pharmacological, molecular, and electrophysiological tools have given conclusion to these hypotheses and have precisely described the role of ion channels in regulating sperm function [2]. Spermatozoa functions such as sperm motility, capacitation, hyperactivity, and chemotaxis are mediated by sperm-specific ion channels [4].

The present mini-review highlights the TRP channels and their potential involvement in regulation of sperm functions. The present review tried to address the functional involvement of TRP channels in regulation of sperm motility, hyperactivity, capacitation, chemotaxis, and thermotaxis. With these objectives, this review searched the database to find the best possible answers related to TRP channel and their function in sperm cells.

## Overview on TRP channels

TRP channels were first reported in *Drosophila melanogaster* mutant by Cosens and Manning [7]. Montell and Rubin [8], after coding of the gene, established the homology of TRP channels with voltage-gated ion channels. TRP channels are a group of unique ion channels that serve as cellular sensors for a wide spectrum of physical and chemical stimuli [9] and constitute a distinct superfamily of ion channels which are distantly related to voltage-gated K^+^ and Na^+^/Ca^2+^ superfamilies [10].

The members of TRP channels are involved in various diverse cellular functions such as vision, taste, hearing, touch, olfaction, thermal perception, and nociception [11]. Many TRP proteins play critical roles in processes such as sensory physiology, vasorelaxation, and male fertility [3]. TRP cation channels are versatile and diverse in their regulatory mechanisms, gating, and selectivity exhibiting a diverse distribution in animal kingdom starting from yeasts, flies, worms, and mice to human. Characterization and nomenclature of TRP channels have been originated from their first members being known as TRP canonical (TRPC) and were involved in phototransduction in *Drosophila* [12]. In sperm cells, events such as capacitation, hypermotility, chemotaxis, and fertilization are mediated by influx of Ca^2+^ and rise in intracellular Ca^2+^ concentration indicating the potential involvement of various types of TRP channels. Thirty different genes of TRP channels have been reported to date and based on sequence similarity; these are divided into following seven groups:

1. TRPC channels
2. Vanilloid TRP channels (TRPV)
3. Melastatin TRP channels (TRPM)
4. Polycystin TRP channels (TRPP)
5. Mucolipin TRP channels
6. Ankyrin TRP channels
7. No mechanoreceptor potential C (nompC) TRP (TRPN) channels: These channels are found in fruit flies and are associated with gating of the channel pore.

The TRP family members are related to signal transduction and are sensitive to pH, temperature, phosphorylation, mechanical, and osmotic changes [13]. The TRPC family has attracted considerable attention due to their putative roles as store-operated and receptor-operated cation channels. In many cells, the emptying of Ca^2+^ stores generates a gating signal that couples intracellular Ca^2+^ release to the opening of store-operated channels in the plasma membrane. Li and Wang [10] reported that TRP channels are responsible for the sustained Ca^2+^ elevations which are necessary for normal physiological functions through store-operated channels [10].

**Figure-1:**
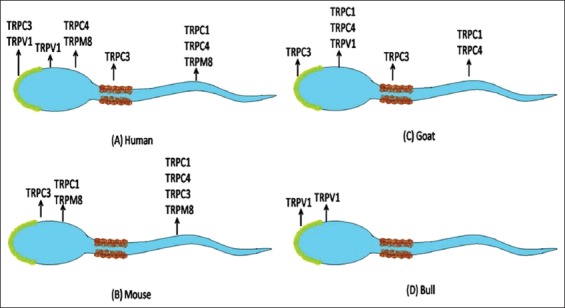
Photomicrograph depicting the functional localization of various types of transient receptor potential (TRP) channels and their subtypes on spermatozoa of various species of animals (A: Human; B: Mouse; C: Goat; and D: Bull). TRPC - TRP canonical; TRPV - TRP vanilloid; TRPM: TRP melastatin (Figure was designed by DKS, AK, and AKM).

TRP expressions are reported in both spermatogenic and mature sperm cells [14]. Protein level organization of TRP channels has been confirmed by X-ray crystallography and electron microscopy [15]. Figure-1 summarizes the various localizations of TRP channels and their various subtypes on various compartments of spermatozoa, thus evidencing their significant role in regulating spermatozoa functions.

### Structure of TRP channels

TRP channels are predicted to have six transmembrane domains with large intracellular amino and carboxyl termini similar to other ion channels [16]. A six-transmembrane helix topology (S1 through S6) with a reentrant loop between S5 and S6 forming the channel pore is a recurring structural motif [17]. These channels tetramerize to a 24-helix functional protein complex. As observed for other ion channels, TRP channel function is strongly influenced by large intracellular domains and the responsiveness to functional modulators, for example, regulation by phosphoinositides [18] or inhibition by quaternary ammonium ions [19] and venom toxins [20]. With this general overview, the present review will mostly focus on understanding the role of different TRP channels and their subtypes in regulating spermatozoa function.

#### TRPC

TRPCs are the first mammalian TRP subfamily discovered in 1995 in *Drosophila* [21]. TRPC proteins are cationic channels and mediate influx of Ca^2+^, Na^+^, and K^+^ on voltage-dependent manner [22]. There are seven TRPC subunits, and they are functionally localized in spermatozoa. Table-1 enlists the different types of TRPC channels in spermatozoa [14,23-28].

**Table-1:** Localization of different types of TRPC on spermatozoa (designed by AK and AKM).

Subtype of TRPC	Localization on spermatozoa	References
TRPC1	Head and flagella of mouse, human, and goat spermatozoa	[24,25]
TRPC2	Anterior part of mouse sperm	[26]
TRPC3 (109 kDa)	Post-acrosomal region and flagella in mouse spermatozoa; acrosomal region and midpiece in human and goat spermatozoa	[14,25,27]
TRPC4	Head and flagella of human and goat spermatozoa and flagella of mouse sperm	[14]
TRPC5 (111 kDa)	Head and flagellum of human sperm	[23]
TRPC6 (140 kDa)	Flagella of mouse, human, goat sperm, and head of human and goat spermatozoa	[14,25]
TRPC7	Not reported in spermatozoa	[28]

TRP=Transient receptor potential, TRPC=Canonical transient receptor potential

Oocyte/egg-sperm interaction involves chemotaxis, capacitation, and hyperactivity of spermatozoa, eventually leading to sperm head fusion and acrosomal reaction (AR) [29]. Studies have shown the involvement of TRPC and its subtype in mediating Ca^2+^ influx. In mouse model, TRPC2 is involved in Ca^2+^ influx while TRPC3 in *Caenorhabditis elegans* [30]. Functional studies have revealed that Ca^2+^ influx is mediated by TRPC2, and in specific, this is triggered by ZP3 [29].

Various localizations of TRPC in spermatozoa have given the indications that they mediate complex series of signaling in spermatozoa, and various compartments are involved in regulating Ca^2+^ influx. Pyr3 which acts as an antagonist of TRPC3 can inhibit sperm motility and accelerate capacitation-associated protein tyrosine phosphorylation in a time- and dose-dependent manner regardless of the presence or absence of Ca^2+^ in the incubation medium [27]. TRPC channel blocker SKF96365 exerted similar effects on mouse sperm motility and capacitation, indicating a potential role of TRPC in mediating sperm motility and capacitation through Ca^2+^ signaling.

#### TRPV

The TRPV channels are constituted by six members activated by physical and chemical stimuli [9]. TRPV is sensitive to changes in pH, osmolarity, ion changes, and high temperature. Various subtypes of TRPV are sensitive to different temperature gradients and get activated to mediate Ca^2+^ influx. TRPV1 to TRPV4 are Ca^2+^ permeable, non-selective cationic channels and are sensitive to endogenous as well as exogenous ligands [31]. TRPV1 is the most studied member of this group, and it is found in urinary bladder [32] and in the reproductive system [33] along with other tissues. Caterina and Julius [34] established the two agonists of this channel that is capsaicin and resiniferatoxin. Anandamide (AEA) is a potent agonist of TRPV1 and also acts as an endovanilloid. Through the activation of TRPV1, AEA reduces mitochondrial activity, and hence, it affects the motility of human sperm. It also inhibits capacitation-induced acrosome reaction [35]. These effects of AEA were prevented by the CB1R antagonist SR141716A, leading to the suggestion that they required CB1R activation while capsazepine promotes spontaneous acrosomal reaction [35] and its use inhibited sperm-oocyte fusion [36]. SR141716A (selective CB1 cannabinoid inverse agonist/antagonist) and SR144528 (selective antagonist of the CB2 cannabinoid receptor) act as potent antagonist of TRPV1 channel [35]. TRPV1 activates endocannabinoid system and is sensitive to high temperature (up to 42°C) and low pH. Its sensitivity is reported to piperine and allicin is modified by the presence of ethanol, nicotine, or changes in phosphatidylinositol 4,5-bisphosphate levels [37].

TRPV2 channel shows 50% similarity in amino acids to TRPV1 and gets activated at approximately 52°C, while TRPV3 and TRPV4 are 40-50% homologous with TRPV1. The activator of TRPV3 is aromatic substances like cloves [38], and temperature ranges from 34 to 39°C [39], while activator of TRPV4 osmotic changes phorbol esters [40] and temperature (25-34°C) [39]. TRPV5 and 6 show the least sensitivity to temperature while very selective to Ca^2+^ and can be modulated by Ca^2+^ [18]. TRPV6 regulates extracellular Ca^2+^ concentration of the epididymal duct which is essential for motility and fertilization capacity of spermatozoa [3]. Table-2 depicts the presence of different types of TRPV on spermatozoa [3,10,35,38,41].

**Table-2:** Localizations of different types of TRPV on spermatozoa (designed by AK and AKM).

Subtype of TRPV	Localization on spermatozoa	References
TRPV1	Acrosomal and post-acrosomal regions of boar, bull, and human spermatozoa	[35,38,41]
TRPV5	Rat spermatozoa (not specified)	[10]
TRPV6	Epididymal epithelium but not in mouse spermatozoa	[3]

TRPV=Vanilloid transient receptor potential

#### TRPMs

TRPM is a member of TRP superfamily and is closely related to the TRPC and TRPV. This subfamily is comprised of eight members (TRPM1 to TRPM8). TRPM1 is first reported in 1998 in tumor suppressor protein melanocytes. TRPM2 as an adenosine diphosphate ribose-activated channel of macrophages; TRPM3 is a hypoosmolarity and sphingosine-activated channel; TRPM4 and TRPM5 are calcium-activated non-selective cation channels; TRPM6 and TRPM7 are magnesium permeable and magnesium-modulated cation channels; and TRPM8 has been described as a cold receptor [42]. TRPM7 is responsible for Zn^2+^ homeostasis in male reproductive tract, and its role is described in sperm maturation [10,42]. TRPM8 is Ca^2+^ permeable non-selective cation channel and is regulated by androgens. It is also associated with Ca^2+^ homeostasis in the prostate epithelium [42,43]. Activation of TRPM8 in capacitated mouse spermatozoa reduced their capacity to undergo the progesterone-induced AR [44]. TRPM8 (approx 130 kDa) channels could be involved in cell signaling events such as thermotaxis or chemotaxis [42,45]. In mouse spermatozoa, TRPM8 detects temperature changes and may influence the AR altering intracellular calcium levels. TRPM8 activation significantly increases [Ca^2+^] and also induces the AR [42,45]. Table-3 depicts the presence of different types of TRPM and their functional localizations on different parts of spermatozoa [10,45,46].

**Table-3:** Localizations of different types of TRPM on spermatozoa (designed by AK and AKM).

Subtype of TRPM	Localization on spermatozoa	References
TRPM3, TRPM4, TRPM5, TRPM6, and TRPM7	Rat spermatogenic cell and spermatozoa	[10,46]
TRPM8	Head and flagellum of mouse and human spermatozoa	[45,46]

TRPM: Transient receptor potential melastatin

#### TRPPs

TRPP subfamily is associated with polycystic kidney disease (PKD) in the human which is under the influence of autosomal dominant gene. This channel is made up of two proteins, namely PKD-1 and PKD-2. PKD-1 is known as TRPP1 and acts as a receptor, while PKD-2 is known as TRPP2 and acts as an ion channel. The PKD2-like subgroup contains three homologous proteins - PKD2, PKD2L1, and PKD2L2 and is referred to as TRPP2, TRPP3, and TRPP5 [25]. Polycystin-1 forms a complex with Polycystin-2 ion channel or protein to regulate various biological processes [47].

PKD and receptor for egg jelly (PKDREJ) expression have been detected only in the mammalian testis, where it is restricted to the spermatogenic lineage and retained in mature sperm. PKDREJ is a specific domain detected only in mammalian testis, head of mature spermatozoa, and spermatogenic lineage [48]. A PKDREJ transcript was detected in spermatogenic cells by *in situ* hybridization with mouse testicular tissue. Polycystin 1 and 2 proteins are detected in the spermatozoa head of the sea urchin and responsible for acrosomal reaction [49]. Human subjects with autosomal PKD due to defects in polycystin (PKD) genes have necrospermia and immotile sperm along with seminal vesicle and ejaculatory duct cysts [50]. TRPP proteins also have been found to be associated with regulation of human sperm motility including other mammals [51]. Table-4 summarizes the localizations of different types of TRPP at different localizations of spermatozoa [25,51].

**Table-4:** Localizations of different types of TRPP on spermatozoa (designed by AK and AKM).

Subtype of TRPP	Localization on spermatozoa	References
TRPP3	Mouse testis; human spermatozoa	[25,51]
TRPP5	Mouse and human spermatozoa	[25,51]

TRPP: Transient receptor potential polycystin

## Conclusion

TRP channels and their various subtypes are involved in regulating spermatozoa functions through regulating calcium homeostasis along with other ion channels. These channels regulate vital functions of spermatozoa such as sperm motility, hypermotility, chemotaxis, capacitation, thermotaxis, and acrosome reaction. TRP channels also make the spermatozoa competent enough to respond to various extracellular stimuli and eventually mediate their function. Understanding the different types of TRPs will help further the science to improve sperm functions as well as will help in the development of suitable measures for the treatment of infertility.

## Authors’ Contributions

AK and AKM designed and framed the manuscript as a part of their research under the supervision of DKS. VS, AS, and SY carried out the proof reading and finalized the manuscript and guided entirely during the preparation of this manuscript. DKS designed the concept and finalized the manuscript for publication. All authors read and approved the final manuscript.

## References

[1] Darszon A, Labarca P, Nishigaki T, Espinosa F (1999). Ion channels in sperm physiology. Physiol. Rev.

[2] Lishko PV, Kirichok Y, Ren D, Navarro B, Chung JJ, Clapham DE (2012). The control of male fertility by spermatozoan ion channels. Ann. Rev. Physiol.

[3] Weissgerber P, Kriebs U (2011). Male fertility depends on Ca^2+^absorption by TRPV6 in epididymal epithelia. Sci. Signal.

[4] Darszon A, Sánchez-Cárdenas C, Orta G, Sánchez-Tusie AA, Beltrán C, López-González I, Granados-González G, Treviño CL (2012). Are TRP channels involved in sperm development and function?. Cell Tissue Res.

[5] Ng KY, Mingels R, Morgan H, Macklon N, Cheong Y (2018). *In vivo* oxygen, temperature and pH dynamics in the female reproductive tract and their importance in human conception: A systematic review. Hum. Reprod. Updat.

[6] Shum WWC, Ruan YC, Da Silva N, Breton S (2011). Establishment of cell-cell crosstalk in the epididymis: Control of luminal acidification. J. Androl.

[7] Cosens DJ, Manning A (1969). Abnormal electroretinogram from a Drosophila mutant. Nature.

[8] Montell C, Rubin GM (1989). Molecular characterization of the Drosophila TRP locus: A putative integral membrane protein required for phototransduction. Neuron.

[9] Zheng J (2013). Molecular mechanism of TRP channels. Compr. Physiol.

[10] Li S, Wang X (2010). Distribution profiles of transient receptor potential melastatin- and vanilloid-related channels in rat spermatogenic cells and sperm. Mol. Biol. Rep.

[11] Latorre R, Zaelzer C, Brauchi S (2009). Structure-functional intimacies of transient receptor potential channels. Q. Rev. Biophys.

[12] Clapham DE (2003). TRP channels as cellular sensors. Nature.

[13] Suh BC, Hille B (2008). PIP2 is a necessary cofactor for ion channel function: How and why?. Ann. Rev. Biophys.

[14] Castellano LE, Trevino CL, Rodriguez D, Serrano CJ, Pacheco J, Tsutsumi V, Felix R, Darszon A (2003). Transient receptor potential (TRPC) channels in human sperm: Expression, cellular localization and involvement in the regulation of flagellar motility. FEBS Lett.

[15] Hellmich RG, Ute A (2014). Structural biology of TRP channels. Mammalian transient receptor potential (TRP) cation channels. Handb. Exp. Pharmacol.

[16] Liao M, Cao E, Julius D, Cheng Y (2013). Structure of the TRPV1 ion channel determined by electron cryo-microscopy. Nature.

[17] She J, Guo J, Chen Q, Zeng W, Jiang Y, Bai XC (2018). Structural insights into the voltage and phospholipid activation of the mammalian TPC1 channel. Nature.

[18] Nilius B, Owsianik G, Voets T (2008). Transient receptor potential channels meet phosphoinositides. EMBO J.

[19] Jara-Oseguera A, Simon SA, Rosenbaum T (2008). TRPV1: On the road to pain relief. Curr. Mol. Pharm.

[20] Siemens J, Zhou S, Piskorowski R, Nikai T, Lumpkin EA, Basbaum AI, King D, Julius D (2006). Spider toxins activate the capsaicin receptor to produce inflammatory pain. Nature.

[21] Zhu X, Chu PB, Peyton M, Birnbaumer L (1995). Molecular cloning of a widely expressed human homolog for the Drosophila TRP gene. FEBS Lett.

[22] Beech DJ (2011). Integration of transient receptor potential canonical channels with lipids. Acta Physiol.

[23] Zhu G, Xie C, Yang Z, Wang Y, Chen D, Wang X (2018). Expression of TRPC5 is decreased in the sperm of patients with varicocele-associated asthenozoospermia. Biomed. Rep.

[24] Darszon A, Nishigaki T, Beltran C, Trevino CL (2011). Calcium channels in the development, maturation and function of spermatozoa. Physiol. Rev.

[25] Kumar PG, Shoeb M (2011). The role of TRP ion channels in testicular function. Adv. Exp. Med. Biol.

[26] Frankenberg S, Schneider NY, Fletcher TP, Shaw G, Renfree MB (2011). Identification of two distinct genes at the vertebrate *TRPC2* locus and their characterisation in a marsupial and a monotreme. BMC Mol. Biol.

[27] Ru Y, Zhou Y, Zhang Y (2015). Transient receptor potential-canonical 3 modulates sperm motility and capacitation-associated protein tyrosine phosphorylation via [Ca^2+^]i mobilization. Acta Biochem. Biophys. Sin.

[28] Numaga T, Wakamori M (2007). TRPC7. Handb. Exp. Pharmacol.

[29] Tosti E, Ménézo Y (2016). Gamete activation: Basic knowledge and clinical applications. Hum. Reprod.

[30] Krauchunas AR, Marcello MR, Singson A (2016). The molecular complexity of fertilization: Introducing the concept of a fertilization synapse. Mol. Reprod. Dev.

[31] Nilius B, Owsianik G, Voets T, Peters JA (2007). Transient receptor potential cation channels in disease. Physiol. Rev.

[32] Szallasi A, Conte B, Goso C, Blumberg PM, Manzini S (1993). Characterization of a peripheral vanilloid (capsaicin) receptor in the rat urinary bladder. Life Sci.

[33] Dorr J, Fecher-Trost C (2011). TRP channels in female reproductive organs and placenta. Adv. Exp. Med. Biol.

[34] Caterina MJ, Julius D (2001). The vanilloid receptor: A molecular gateway to the pain pathway. Ann. Rev. Neurosci.

[35] Gervasi MG, Osycka-Salut C (2011). Anandamide capacitates bull spermatozoa through CB1 and TRPV1 activation. PLoS One.

[36] Francavilla F, Battista N (2009). Characterization of the endocannabinoid system in human spermatozoa and involvement of transient receptor potential vanilloid 1 receptor in their fertilizing ability. Endocrinology.

[37] Rizopoulos T, Papadaki-Petrou H, Assimakopoulou M (2018). Expression profiling of the transient receptor potential vanilloid (TRPV) channels 1, 2, 3 and 4 in mucosal epithelium of human ulcerative colitis. Cells.

[38] Xu H, Delling M (2006). Oregano, thyme and clove-derived flavors and skin sensitizers activate specific TRP channels. Nat. Neurosci.

[39] Jeong JH, Lee DK, Liu SM, Chua SC, Schwartz GJ, Jo YH (2018). Activation of temperature-sensitive TRPV1-like receptors in ARC POMC neurons reduces food intake. PLoS Biol.

[40] Watanabe H, Davis JB (2002). Activation of TRPV4 channels (hVRL-2/mTRP12) by phorbol derivatives. J. Biol. Chem.

[41] Bernabo N, Pistilli MG (2010). Role of TRPV1 channels in boar spermatozoa acquisition of fertilizing ability. Mol. Cell. Endocrinol.

[42] Zierler S, Hampe S, Nadolni W (2017). TRPM channels as potential therapeutic targets against pro-inflammatory diseases. Cell Calcium.

[43] Yang Z, Wang X, Zhu G, Zhou Z, Wang Y, Chen D, Meng Z (2012). Effect of surgical castration on expression of TRPM8 in urogenital tract of male rats. Mol. Biol. Rep.

[44] Gibbs GM, Orta G (2011). Cysteine-rich secretory protein 4 is an inhibitor of transient receptor potential M8 with a role in establishing sperm function. Proc. Natl. Acad. Sci. U. S. A.

[45] De Blas GA, Darszon A (2009). TRPM8, a versatile channel in human sperm. PLoS One.

[46] Martinez-Lopez P, Trevino C (2011). TRPM8 in mouse sperm detects temperature changes and may influence the acrosome reaction. J. Cell. Physiol.

[47] Sutton KA, Jungnickel MK (2006). Functional characterization of PKDREJ, a male germ cell-restricted polycystin. J. Cell. Physiol.

[48] Butscheid Y, Chubanov V, Steger K, Meyer D, Dietrich A, Gudermann T (2006). Polycystic kidney disease and receptor for egg jelly is a plasma membrane protein of mouse sperm head. Mol. Reprod. Dev.

[49] Neill AT, Vacquier VD (2004). Ligands and receptors mediating signal transduction in sea urchin spermatozoa. Reproduction.

[50] Vora N, Perrone R, Bianchi DW (2008). Reproductive issues for adults with autosomal dominant polycystic kidney disease. Am. J. Kidney Dis.

[51] Sutton KA, Jungnickel MK (2008). A polycystin-1 controls postcopulatory reproductive selection in mice. Proc. Natl. Acad. Sci. U. S. A.

